# Management of a Pregnant Woman With Malaria, Stillbirth, and a 12 × 11-cm Amoebic Liver Abscess in a Burundian Hospital: A Case Report

**DOI:** 10.1155/crog/9403522

**Published:** 2025-08-30

**Authors:** Maria Antonietta Castaldi, Salvatore Giovanni Castaldi

**Affiliations:** ^1^Department of Medicine, Surgery and Dentistry “Scuola Medica Salernitana”, University of Salerno, Baronissi, Salerno, Italy; ^2^Department of Obstetrics and Gynecology, Hôpital Autonome de Ngozi, Ngozi, Burundi

**Keywords:** amoebic abscess, Burundi, malaria, pregnancy, stillbirth

## Abstract

**Background:** Malaria and amoebic infections are considered risk factors for stillbirth and preterm labor, but their coexistence during pregnancy has not been previously reported.

**Case:** We describe the first case of averted maternal mortality with fetal death in utero at 22 weeks' gestation, complicated by both *falciparum* malaria and hepatic amoebic abscess, in a rural hospital in Burundi.

**Conclusion:** Amoebic liver abscesses are rarely described in pregnancy and, as far as we are aware, never in conjunction with severe malaria: two parasitic infections requiring completely different treatments. We successfully managed this difficult case in a rural context thanks to at least three main factors: staff skills, inventiveness, and advances in ultrasonographic technology.

**Summary**

• Successful management of a stillbirth in a woman who developed severe malaria and hepatic amoebic abscess during pregnancy in a rural hospital in Burundi.

## 1. Introduction

In 2015, Burundi recorded a maternal mortality rate of 1000 per 100,000 live births, ranking among the highest in Africa and reflecting one of the continent's most critical public health challenges [1, 2]. Maternal mortality and morbidity remain the leading contributors to disease burden in Burundi [3]. The persistently poor maternal health outcomes are largely due to limited access to and underutilization of essential health services. In this context, health authorities have identified malaria and amoebiasis as two of the primary causes of maternal deaths in this country [1, 4].

In particular, malaria and amoebic disease in pregnancy represent a serious health problem [1, 4–7], as pregnancy lowers both native and specific immune responses [8, 9]. This is thought to be due to increased progesterone levels during pregnancy, which are responsible for decreased NK cell numbers, an altered Th1/Th2 balance, and the failure of immunoglobulin levels to rise in infections [8–11].

The effects of malaria in pregnancy include elevated risks of maternal anemia, preterm birth, congenital malaria, and low birth weight (LBW), as well as possible outcomes such as spontaneous abortion, stillbirth [12], and maternal mortality [13].

Amoebic dysentery in pregnancy has been extensively reported, whereas cases of amoebic liver abscess (ALA), the most common extraintestinal manifestation of *Entamoeba histolytica* infection, are comparatively rare [6]. Diagnosis of ALA is often challenging in developing countries due to the nonspecific clinical presentation and limited access to appropriate medical resources. Pregnancy is considered a risk factor for the development of invasive amoebiasis, including ALA, due to immunological and hormonal changes that increase susceptibility [14–16]. Amoebic infection during pregnancy is associated with an increased risk of intrauterine growth restriction (IUGR), preterm labor, and LBW and may be linked to fetal demise [6, 17].

In this study, the management of a stillbirth case in a pregnant woman with both malaria and ALA in a rural Burundian hospital is described.

## 2. Case Report

A 29-year-old primigravida with fetal demise presented at the Emergency Obstetric Care Unit of Hôpital Autonome de Ngozi, Ngozi, Burundi. Upon admission, she was pyrexial (38°C), clinically septic, hypotensive (85/55 mmHg), and tachycardic, with a respiratory rate of 22 breaths per minute, a heart rate of 120 bpm, and a shock index of 1.4 [18]. Additionally, she was hypoglycemic (57 mg/dL), with a Glasgow Coma Scale (GCS) score of 14. The patient was 158-cm tall, weighed 48 kg, with a BMI of approximately 19.2.

The patient presented with generalized abdominal tenderness and had been in spontaneous labor since the previous day. An emergency cesarean section (CS) was performed due to prolonged labor, with a closed internal cervical OS and marked hemodynamic instability attributable to sepsis. A demised fetus at 22 weeks of gestation, as determined by the last menstrual period (LMP), was delivered. No signs of ascites or other intra-abdominal pathology were observed during the CS.

After CS, the woman was transferred to the local intensive care unit (ICU) of the hospital due to respiratory and hemodynamic instability. She was pyrexial, confused, and presented with severe hypotension (BP immeasurable) and hypoglycemia (40 mmHg). The hemoglobin color scale (HbCS, showed a hemoglobin level of 6.5 g/dL (Copack GmbH, Germany) [19]. In addition, due to the poor and rural conditions of the hospital, no hemoculture or other laboratory parameters were available to better define the septicemic state of the patient.

Medical therapy began with dopamine (initial infusion rate of 5 *μ*g/kg/min, increasing at need) and ampicillin (1000 mg, IV) every 8 h, gentamicin (1 mg/kg) every 8 h, oxygen (4 L/min), and 50% glucose solution. Based on an initial clinical suspicion of malaria, quinine treatment was initiated, diluted in 500 mL of 5% glucose solution, with a loading dose of 20 mg/kg administered over 4 h, followed by a maintenance dose of 10 mg/kg every 8 h. Malaria was confirmed by the identification of *Plasmodium falciparum* upon microscopic examination of Giemsa-stained thick blood smear [20, 21], and quinine treatment was subsequently continued. No other pharmacological options, such as artesunate or artemether, were available at the Hôpital Autonome de Ngozi. The patient was also tested for HIV, and the result was negative.

**Figure 1:**
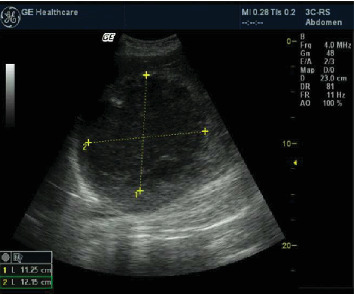
The 11 × 12-cm collected amoebic liver abscess (outlines) with regular and well-defined rims, and an echoic dense and homogeneous content, located in the right upper quadrant.

The day after her CS, the patient's condition improved, but she complained of right upper quadrant pain and substantial hepatic tenderness. An ultrasonographic exam was performed, revealing an 11 × 12-cm liver abscess located in the right upper quadrant (Figure 1). The sonographic pattern was described as a collected ALA according to the N'Gbesso criteria [17].

Thus, we decided to treat the abscess via percutaneous drainage under sonographic guidance. The percutaneous treatment procedure was performed under local anesthesia (2% lidocaine) and continuous real-time sonographic guidance using the freehand technique. For sonographic guidance, a portable device (LOGIC Book XP; General Electric Medical Systems, Milwaukee, WI) and a 4-MHz convex transducer were used. An 18-gauge needle was used to aspirate the abscess cavity, and 200 mL of “anchovy paste” was drained from the abscess. Repeated washings with physiological solution were performed, and local metronidazole was instilled in the abscess cavity. Therapy with systemic metronidazole was initiated at a dose of 500 mg every 8 h; when microscopy of the pus revealed *Entamoeba histolytica*, although no organism was cultured, metronidazole was continued. To drain the remaining 500 mL of liquid, repeated percutaneous drainage was performed the day after, with the same settings.

On the third day after her recovery in the ICU, the patient's condition deteriorated with marked hypotension, hypoglycemia, and a semiconscious state. Therapy with dopamine was restarted, but it was ineffective. HbCS showed a hemoglobin level of 5 g/dL, likely due to a hemolytic crisis secondary to malaria infection. Thus, we decided to perform a blood transfusion; however, neither blood nor colloid was available, so therapy with two units of frozen plasma was set. Moreover, as there was no 50% glucose solution, three repeated infusions with 10% glucose solution were ordered to improve glycemic balance.

On the fourth day, BP was stable at 105/60 mmHg, glycemic profile was 80 mg/100 mL, and the patient was conscious. Paracentesis was performed to remove 1000 mL of straw-colored peritoneal fluid. Bedside abdominal ultrasound was used both pre- and intraoperatively to confirm the presence and distribution of the fluid. The ascites was likely reactive, associated with the underlying septic state and systemic inflammatory response.

Antibiotic, antimalarial, and antiamoebic therapy was continued for the following days. Thereafter, the patient left the ICU with a favorable outcome.

## 3. Discussion

This patient had two confirmed parasitic infections, complicating a pregnancy of 22 weeks' gestation, presenting with fetal loss.

Hormonal fluctuations—particularly in estradiol, progesterone, and gonadotrophic hormones—play a crucial role in modulating the immune system to prevent fetal rejection during pregnancy [10]. Maternal immune tolerance is maintained through local and systemic mechanisms, largely regulated by sex hormones [10, 11]. Progesterone, via the progesterone-induced blocking factor (PIBF), suppresses natural killer (NK) cell activity and promotes a Th2-dominant immune response [8]. This shift alters humoral immunity, increasing susceptibility to parasitic infections such as malaria and amebiasis, which can become clinically apparent during pregnancy [8, 10].

Therefore, malaria infection during pregnancy is a serious health problem with serious repercussions for both mother and fetus in most of the world's tropical regions [22]. Indeed, even among women in sub-Saharan Africa, who typically possess a high level of immunity to malaria, this immunity is altered during pregnancy [23].

The major adverse effect of malaria during pregnancy is maternal anemia [24], followed by cerebral malaria, hypoglycemia, and pulmonary edema [23].

Malaria during pregnancy is a common cause of LBW babies [25]; likewise, congenital infection, possibly spontaneous abortion, and stillbirth have also been documented [13].

However, in its invasive form, *Entamoeba histolytica* is responsible for clinical syndromes, ranging from classical dysentery to extraintestinal disease with emphasis on hepatic amebiasis [26]. Indeed, ALA is the most common extraintestinal manifestation of infection with *Entamoeba histolytica*, and it is associated with significant morbidity and mortality [26]. Furthermore, cardiovascular and respiratory problems, metabolic disturbances, renal failure, and shock are all described in amoebic disease [27], with amoebic dysentery and liver abscesses in pregnancy also reported [6].

Moreover, amoebic disease can cause preterm labor, IUGR, and LBW, and a correlation with abortion has also been described [6, 17, 28].

In this study, the management of a stillbirth case in a pregnant woman with both malaria and ALA in a rural Burundian hospital is described. The Hôpital Autonome de Ngozi, located in Ngozi Town in northern Burundi, serves as a key public referral center for approximately 120,000 people in the Ngozi Health District, alongside the faith-based Mivo Hospital [29]. Established in 1930, this hospital offers a broad range of medical services, including emergency care, surgery, internal medicine, pediatrics, laboratory diagnostics, physiotherapy, and maternity care. However, like many healthcare facilities in low-resource settings, this hospital faces significant challenges, such as overcrowding, inadequate bed capacity (occasionally resulting in patients sleeping outdoors), staff shortages (particularly among nursing personnel), and recurrent shortages of essential medical supplies and equipment [29]. These infrastructural and resource constraints complicate the delivery of quality maternal and neonatal healthcare, highlighting the urgent need for increased investment and support to improve health outcomes in this and similar settings.

The present case was recorded during a healthcare mission to Burundi undertaken by the principal author, and to our best knowledge, this is the first reported case of stillbirth in a woman who developed severe malaria and hepatic amoebic abscess during pregnancy; this will allow us to reconsider the burden of such diseases on pregnancy.

The combination of malaria and a hepatic amoebic abscess has never been described previously in pregnancy, and as malaria and amoebas are both implicated in abortion and/or preterm labor, we hypothesize that the loss of pregnancy in the present case could be due to a combination of the two pathological conditions. Indeed, the passage of parasites and their antigens across the placenta occurs with metazoan and protozoan parasites [30]. Additionally, a previous study reported that microscopic screening of the cord blood of newborns positive for amoebas or malaria at delivery showed a parasite rate of 10.95%, and transplacental passage of *Plasmodium falciparum* and *Entamoeba histolytica* was confirmed by parasitemia in the peripheral blood of 2.82% of newborns within 7 days of birth [30].

In our case, although it was not possible to reveal the presence of parasites because of the rural context, based on the international literature, we believe that the transplacental passage of both *Plasmodium falciparum* and *Entamoeba histolytica* caused the fetal death, possibly in combination with maternal anemia.

Additionally, the combination of these two infections may have contributed to our patient's difficult postoperative recovery when compared with previous cases described for pregnancy [28].

Indeed, after the emergency CS, the patient's metabolic, cardiovascular, and respiratory conditions dramatically worsened, and she was suddenly moved to the ICU. Her metabolic state was thus significantly compromised: Severe hypoglycemia has been present since her recovery.

Indeed, since malaria is well known to cause hypoglycemia [23], and ALA is described to provoke metabolic disturbances [27], in our case, this combination resulted in intractable hypoglycemia even after many attempts. Moreover, on the third day of recovery, the treatment of the hypoglycemia became much more difficult using a 10% glucose solution, the only option available, but this was necessary to save the patient's life. The excessive peritoneal fluid was anticipated, so paracentesis was performed, as diuretics could not be used because of the patient's hypotensive state.

Partially due to her debilitated state and malaria (anemia), the patient's hypotension, present since her initial recovery, worsened with further blood loss during CS. Notably, cardiovascular disturbances and shock have both been reported for amoebic disease [27]. At first, this severe hypotension was successfully treated with dopamine infusion, but her worsening condition on the third day after her recovery in the ICU necessitated a blood transfusion after dopamine failure. Nevertheless, the lack of blood forced us to turn to frozen plasma.

Furthermore, after CS, the woman was dysphonic and cyanotic, so she was treated with oxygen (4 L/min) to aid her breathing. These breathing alterations are well explained by her severe anemia, caused both by malaria and blood loss during the surgical intervention, as well as possible respiratory disease occurring because of the amoebic disease.

Currently, special focus on ALA treatment is needed to better understand how technological advances may help to save lives even in the developing world.

The day after her CS, the patient improved but presented symptoms of ALA: fever, right upper quadrant pain, and substantial hepatic tenderness. An ultrasonographic examination confirmed the presence of a 12 × 11-cm abscess, which seemed to be a collected ALA according to the N'Gbesso classification; indeed, a hepatic scan showed regular and well-defined rims and echoically dense and homogeneous content [17]. Early percutaneous drainage was chosen because it may improve symptoms and shorten hospital stays for collected abscesses larger than 5 cm [31, 32]. Furthermore, in recent years, imaging-guided percutaneous treatment has replaced surgical intervention as the primary treatment for liver abscesses [33]. Two possible methods are available for this treatment: needle aspiration and catheter drainage. The main advantages of needle aspiration over catheter drainage are as follows: needle aspiration is less invasive and less expensive; it avoids problems related to follow-up catheter care, so less medical or nursing care is required; and multiple cavities can be aspirated in the same session [34]. Moreover, these advances in ultrasonographic technology were essential in our case: Indeed, the sonographic guidance was performed with a portable device. In addition, the instillation of metronidazole directly into an abscess cavity has never been reported in the literature, thus representing a unicum.

Our decision to start local and systemic therapy with metronidazole was based on the ultrasonographic results and the aspiration of “anchovy paste,” which is typically referred to as amoebic [35]. The microscopic exam also confirmed our diagnosis [27].

In conclusion, on the one hand, the burden of malaria and amoebas on stillbirth should be reconsidered; on the other hand, we successfully managed this difficult case in a rural context thanks to at least three main factors: staff skills, inventiveness, and advances in ultrasonographic technology.

## Data Availability

The data that supports the findings of this study are included within the article itself.
